# Induction of Axillary Bud Swelling of *Hevea brasiliensis* to Regenerate Plants through Somatic Embryogenesis and Analysis of Genetic Stability

**DOI:** 10.3390/plants12091803

**Published:** 2023-04-28

**Authors:** Taihua Wang, Jinu Udayabhanu, Xiaochuan Gu, Rizhi Wu, Shichao Xin, Qiuhui Chen, Yuanyuan Zhang, Xianfeng Yang, Suna Peng, Jianmiao Chen, Tiandai Huang

**Affiliations:** 1 Sanya Nanfan Research Institute of Hainan University, Hainan Yazhou Bay Seed Laboratory, Sanya 572025, China; 13398955764@163.com; 2 College of Tropical Crops, Hainan University, Haikou 570228, China; 3 Rubber Research Institute, Chinese Academy of Tropical Agricultural Science, Haikou 571101, China; jinu.v.bhanu@gmail.com (J.U.); guxiang28@163.com (X.G.); wrz185@163.com (R.W.); scxin@catas.cn (S.X.); cqhluck@163.com (Q.C.); zhangyuanyuan@catas.cn (Y.Z.); shouyiweida@163.com (X.Y.); pengsuna2013@163.com (S.P.); 4 Haikou Key Laboratory of Innovation of Seedlings of Tropical Plants, Haikou 571101, China

**Keywords:** *Hevea brasiliensis*, axillary bud, somatic embryo, regeneration, genetic stability

## Abstract

To overcome rubber tree (RT) tissue culture explant source limitations, the current study aimed to establish a new *Hevea brasiliensis* somatic embryogenesis (SE) system, laying the technical foundation for the establishment of an axillary-bud-based seedling regeneration system. In this study, in vitro plantlets of *Hevea brasiliensis* Chinese Academy of Tropical Agricultural Sciences 917 (CATAS 917) were used as the experimental materials. Firstly, the optimum conditions for axillary bud swelling were studied; then, the effects of phenology, the swelling time of axillary buds (ABs), and medium of embryogenic callus induction were studied. Plantlets were obtained through somatic embryogenesis. Flow cytometry, inter-simple sequence repeat (ISSR molecular marker) and chromosome karyotype analysis were used to study the genetic stability of regenerated plants along with budding seedlings (BSs) and secondary somatic embryo seedlings (SSESs) as the control. The results show that the rubber tree’s phenology period was mature, and the axillary bud induction rate was the highest in the 2 mg/L 6-benzyladenine (6-BA) medium (up to 85.83%). Later, 3-day-old swelling axillary buds were used as explants for callogenesis and somatic embryogenesis. The callus induction rate was optimum in MH (Medium in Hevea) + 1.5 mg/L 2,4-dichlorophenoxyacetic acid (2,4-D) + 1.5 mg/L 1-naphthalene acetic acid (NAA) + 1.5 mg/L Kinetin (KT) + 70 g/L sucrose (56.55%). The regenerated plants were obtained after the 175-day culture of explants through callus induction, embryogenic callus induction, somatic embryo development, and plant regeneration. Compared with the secondary somatic embryo seedling control, axillary bud regeneration plants (ABRPs) were normal diploid plants at the cellular and molecular level, with a variation rate of 7.74%.

## 1. Introduction

*Hevea brasiliensis* belongs to the genus Hevea of the Euphorbiaceae family; it is a perennial cross-pollinated tree. The natural rubber it produces is a strategic industrial raw material, ideal for manufacturing aircraft and automobile tires, and has the advantages of high strength, good flexibility, and high wear resistance [1]. Advanced tissue culture methods have enabled the large-scale propagation of Hevea seedlings, also leading to the creation of genetically modified clones with high yield and various disease- or pest-resistant cultivars [2]. SE has received more attention for the mass production of Hevea tissue culture seedlings. Recently, transgenic research on Hevea has considerably explored the possibilities of somatic embryogenesis for the development of transgenic lines that are resistant to various biotic and abiotic challenges.

A number of in vitro approaches, such as anther culture [3], inner-integument culture [4], the regeneration of the fragile embryogenic callus [5], and secondary embryo circulatory proliferation [6] are being implemented in Hevea tissue culture for the regeneration of plantlets through somatic embryogenesis. Various researchers have already reported the future prospects of primary and secondary embryogenesis for regenerating Hevea seedlings on a commercial level [7]. Transcriptional-level studies of SE in Hevea were conducted by [8] for an actual understanding of molecular regulation during the rubber tree’s late SE.

Due to the seasonal nature of main donor explants, anthers, and inner-integument availability, the regeneration system is easily restricted by season and time, making it impossible to achieve year-round cultivation. Anther culture is limited by time, with only three flowering stages: spring, summer, and autumn flowers. The inner-integument sampling time is generally 45 to 75 days after natural pollination. In addition, for the secondary embryo cycle proliferation system, the primary embryo is derived from the anther or inner integument, which are also limited by the seasons. The above problems can be overcome by choosing the new explant ABs of *Hevea brasiliensis*. In other crops such as cassava (*Manihot esculenta* Crantz) [9], banana (*Musa nana* Lour.) [10], poplar (*Populus* L.) [11], ginkgo (*Ginkgo biloba* L.) [12], and (*Primula × pubescens*) [13], an AB-based tissue culture system is a viable method for seedling breeding. Therefore, we must study the potential of *Hevea brasiliensis* ABs for the seedling regeneration system through SE.

There are few studies on plant regeneration using RT ABs as explants. In the RT, adventitious buds were induced by stem tips and successfully regenerated seedlings with a rapid growth rate and no deformity [14]. In another study, the leaves and ABs of *Hevea brasiliensis* were used as explants to induce plant regeneration, and the callus induction rate of ABs was higher than that of leaves. ABs could directly induce small buds and developed into a good root system; however, they were still unable to induce somatic embryos successfully [15]. Using the ABs of *Hevea brasiliensis* as explants, regenerated plants were induced by the callus and somatic embryo; however, there was no detailed picture description [16]. Thus, we designed the current study to give in-depth information about the possibility of AB swelling as a source plant for the complete callogenesis and somatic embryogenesis pathway for the large-scale regeneration of Hevea seedlings. We also analyzed the genetic stability of the regenerated plants to determine the genomic mechanism of the explant source to establish a plant regeneration system in Hevea. Thus, our study provides a technical reference for the production of in vitro seedlings as well as the transgenic plants of Hevea self-rooted juvenile clones in large-scale propagation.

## 2. Results

### 2.1. Effects of Different Influencing Factors on AB Swelling

#### 2.1.1. Effect of Phenology on AB Swelling

**Figure 1:**
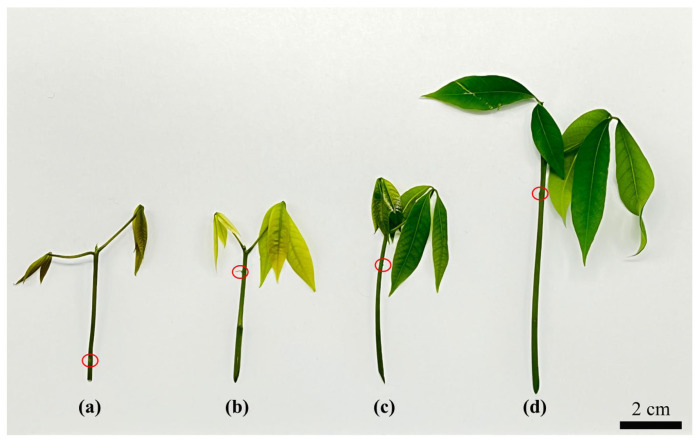
Phenological classification of RT in vitro plantlets. (**a**) Bronze period; (**b**) color-change period; (**c**) pale-green period; (**d**) mature period. Note: Red circle shows that ABs could grow from nodes.

After dissecting 1 cm-long stem segments from seedings under different phenological levels, they were inoculated in the M1 Medium. After 3~4 d of inoculation, the ABs began to bulge on the stem segment. The swelling rate was different in each phenological level under the same growth conditions. In Table 1, the effect of the phenological period on AB swelling was significantly different, and the swelling rate of ABs on stem segments in the mature period was significantly higher than in the color-change period and pale-green period; however, there was no significant difference between the ability of ABs to swell in the bronze period or the mature period. The ability of ABs to swell was ranked in descending order: mature period, bronze period, pale-green period, and color-change period (Figure 1).

#### 2.1.2. Effect of 6-BA Concentration on AB Swelling

Under the conditions of different 6-BA concentrations, the AB swelling rate was significantly higher at 2 mg/L and 1.2 mg/L than in the control group (0.0 mg/L 6-BA). No significant difference was observed between the other two concentrations (1.6 mg/L and 0.4 mg/L). In total, 0.8 mg/L showed the lowest level of AB swelling. The results are tabulated in Table 2. The swelling rate was not completely concentration-dependent, despite 6-BA at 2 mg/L providing an excellent number of AB swellings (85.83 ± 5.20).

### 2.2. Effects of Different Factors on the Induction of AB Callus

#### 2.2.1. Effect of AB Swelling Time on Callus Induction

**Figure 2:**
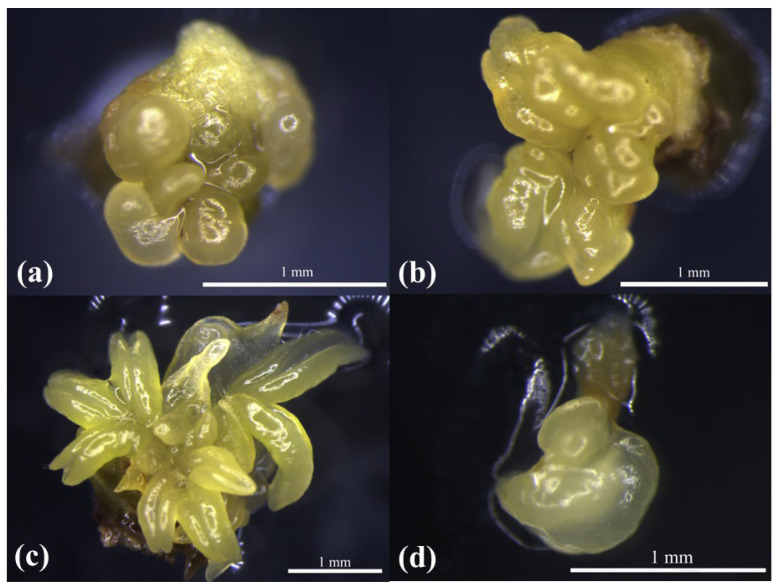
Morphological observation of AB callus with different swelling times: (**a**) 3 d AB callus growth was large and granular; (**b**) irregular morphology of AB callus after 6 d; (**c**) 10 d ABs tended to elongate, and almost no callus occurred; (**d**) the callus of differentiated young leaves was less pronounced.

After inoculating stem segments (from the mature period) with ABs in the M1 medium, different levels of AB swellings and differentiated tender leaves were obtained at different time durations. Later, the callus was induced in the M2 medium, and the average callogenesis induction rate was tabulated (Table 3). The highest average AB callus induction rate was observed at 3 d (Figure 2a) and 6 d (Figure 2b) in the AB swellings compared with the 10 d (Figure 2c) AB swellings and differentiated tender leaves (Figure 2d). The average occurrence rate of the AB callus with a swelling time of 6 d was higher than that of 3 d; however, the status and quality of the differentiated callus were not as good as those of the ABs with a swelling time of 3 d. The ABs with swelling time of 10 d had almost no callus differentiation, reflected in the elongation of buds. Similarly, a small amount of callus was obtained from differentiated young leaves. Considering the results obtained, the 3 d AB induction was the optimum for callogenesis.

#### 2.2.2. Histological Analysis

**Figure 3:**
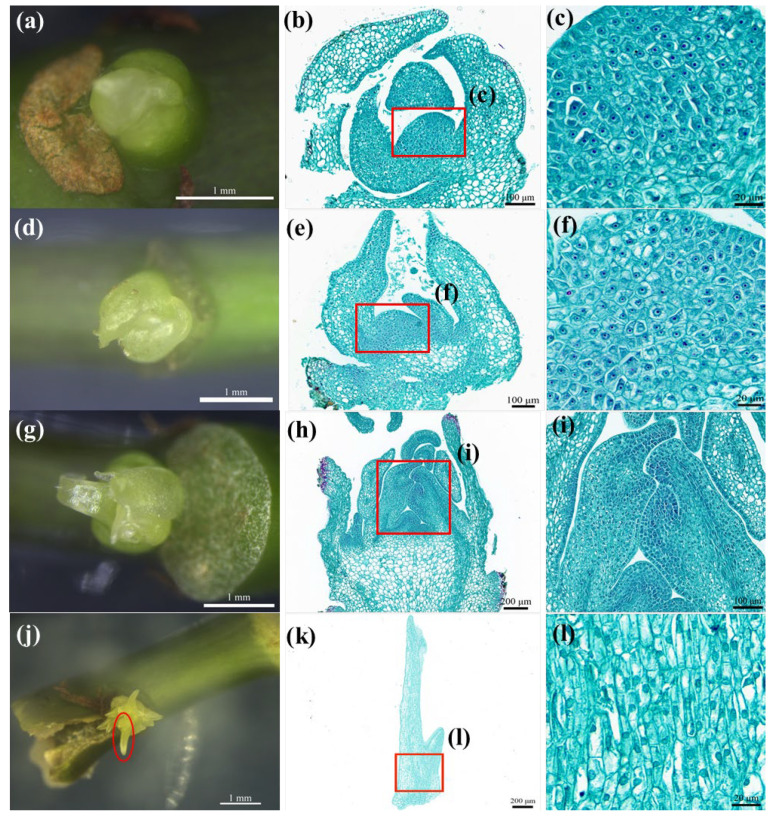
Morphological observations and paraffin sections of ABs and differentiated young leaves at different swelling times. (**a**,**d**,**g**) The ABs swelled in M1 medium for 3 d, 6 d, and 10 d, respectively; (**j**) the red circle is the differentiated young leaves; (**b**,**e**,**h**,**k**) correspond to the histological observations of explants in (**a**,**d**,**g**,**h)**, respectively; (**c**) 3 d ABs had a vigorous meristem ability; (**f**) the meristematic cells of 6 d ABs were not as dense as those of 3 d ABs; (**i**) 10 d ABs showed a differentiation of young leaves; (**l**) there were a few meristematic cells in the differentiated young leaves (The red boxes in (**b**,**e**,**h**,**k**) are enlarged portion of (**c**,**f**,**i**,**l**), respectively).

Based on the morphological observations of ABs with different swelling times, we found that 3 d-old ABs (Figure 3a) were at the beginning of swelling. The histological section showed (Figure 3b) that the meristem cells located in the center of the ABs had an obvious nucleus, dense cytoplasm, and a strong meristem ability (Figure 3c). Morphologically, 6 d-old AB swelling (Figure 3d) was bigger than the 3 d swelling. The paraffin sections showed that the intercellular arrangement of 6 d-old ABs was usually characterized by tender leaf differentiation (Figure 3e), with meristem cells’ cytoplasm being less dense than that of 3 d-old ABs (Figure 3f). In the ABs with a swelling time of 10 d (Figure 3g), the young leaves began to elongate at the center (Figure 3h). The differentiated young leaves (Figure 3j) showed a small amount of meristem (Figure 3l). Table 3 shows that the mean callus induction rate of the ABs with a 3 d swelling time was not significantly different from that of the 6 d ABs, and the 3 d ABs had more cytoplasmic-dense meristem cells. In conclusion, it is best to select explants during the 3 d swelling time of ABs in M1.

#### 2.2.3. Effect of Basic Medium on AB Callus Tissue Induction

**Figure 4:**
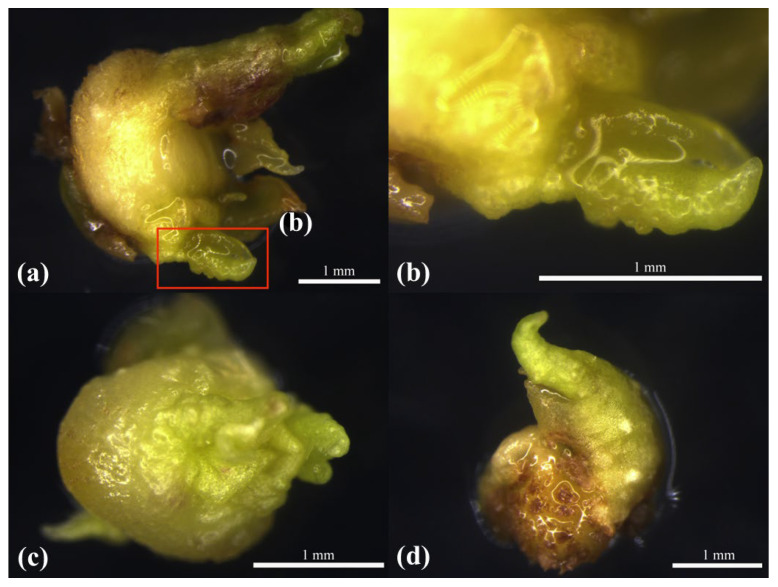
Morphological observation of ABs in MH, MS, and WPM media at 3 d: (**a**) 3 d ABs had a tendency to differentiate in basic MH medium; (**b**) enlargement of the area in the red box in (**a**); (**c**) 3 d ABs in basic MS medium overall expansion; (**d**) WPM basic medium elongation hardening.

The basic MS medium had a considerable influence on the growth of ABs compared with NN [18], NLN medium [19], N6 medium [20], Gamborg’s medium (B5) [21], GD [22], and SH [23] medium. The ABs of the seven basic media grew slowly and browned considerably, with a low survival rate and showing signs of expansion and elongation; however, they generally became hard and old. Woody Plant Medium (WPM) [24] quickened the growth; however, only elongation without differentiation occurred, and its surface became dry and hard. The MS medium resulted in almost no browning, with an overall expansion and no elongation. The MH medium had the highest survival rate among the ABs, with low browning, overall expansion without elongation, and good AB growth, with a fresh, non-drying surface, tissue protrusion, and a tendency to differentiate. The results are tabulated (Table 4) and recorded (Figure 4).

#### 2.2.4. Effects of Plant Growth Regulators on AB Callus Induction

**Figure 5:**
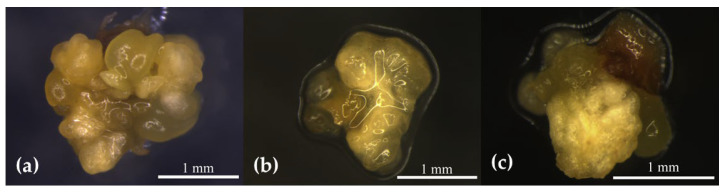
Morphological observation of AB callus under different plant growth regulators. (**a**) The 3 d AB callus reaction under No. 1 treatment was obvious, and the callus grew more; (**b**,**c**) the growth of the 3 d AB callus under No. 6 and No. 8 treatments was less.

Three-day-old ABs were cultured for thirty days in callus-induction medium with nine different hormone concentrations, and the average callus incidences were significantly different (Table 5). The mean callus incidence was significantly higher in the No. 1 media than the other 8 media (2, 3, 4, 5, 6, 7, 8 and 9). Moreover, the callus in the No. 1 medium exhibited slight browning and high growth (Figure 5a); therefore, it was considered the best.

An intuitive analysis showed that KT had the greatest effect on the induction of the callus, followed by sucrose, while 2,4-D and NAA had less of an effect (Table 6). The four influencing factors on callus induction were, in descending order, KT > sucrose > 2,4-D > NAA. For callus induction, the best hormone combination was KT 1.5 mg/L + 2,4-D 1.5 mg/L + NAA 1.5 mg/L + sucrose 70 g/L, i.e., medium No. 1.

### 2.3. Establishment of AB Regeneration System of Sterile RT

**Figure 6:**
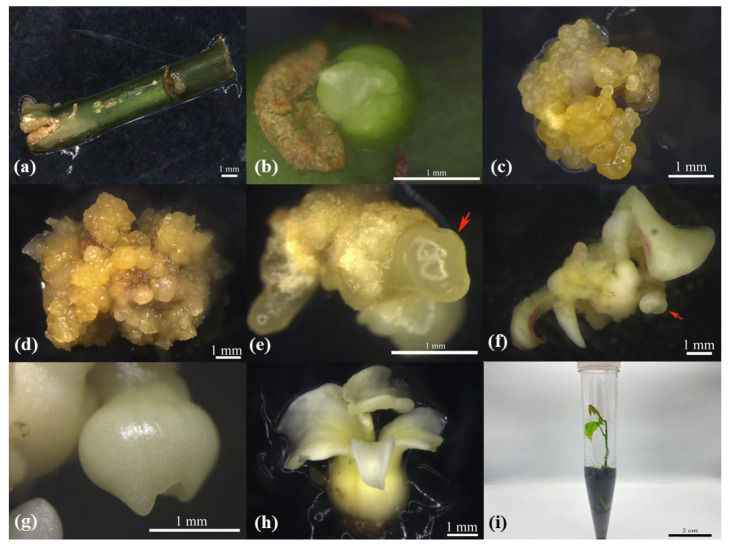
Process of AB regeneration system of in vitro plantlets of RT CATAS 917. (**a**) Mature-period stem segment is 1 cm-long with one bud point; (**b**) ABs with 3 d swelling time; (**c**) callus tissue; (**d**) clumps of embryogenic and non-embryogenic callus; (**e**) globular embryo; (**f**) heart-shaped embryo; (**g**) torpedo embryo; (**h**) mature cotyledon somatic embryo; (**i**) a well-grown regenerated plant. (The arrows (**e**,**f**) are shown as the same parts).

From the mature in vitro plantlets, 1 cm-long stem segments with one bud point (Figure 6a) were dissected out and inoculated in M1, and AB swellings were obtained after 3 days of inoculation. The 3 d-old ABs (Figure 6b) were taken as explants and inoculated in M2 for 25~30 d for callus induction (Figure 6c). Clumps of embryogenic callus and non-embryogenic callus (Figure 6d) were induced in the further inoculation in M3 for 25~30 d, and they were later transferred to M4 for 13~15 d to induce the formation of sphere-like embryos (Figure 6e), before being subsequently subcultured for 21~27 d, resulting in heart-shaped embryos (Figure 6f). In the next 10 d, torpedo-shaped embryos developed (Figure 6g), which then developed into cotyledon embryos (Figure 6h) in the next 25~30 d. Later, they were transferred to M5 medium for plantlet regeneration. It took up to 40–45 d for the complete growth and development of plantlets (Figure 6i). In brief, ABs with a swelling time of 3 d were selected as explants, and callus tissue with somatic embryogenesis was obtained at 60 d. Then, mature cotyledon-shaped embryos with regeneration capacity could be obtained at 135 d, and regenerated plants with good growth could be obtained at 175 d.

### 2.4. Analysis of the Genetic Stability of the Regenerated Plants

#### 2.4.1. Chromosomal Karyotype Analysis

**Figure 7:**
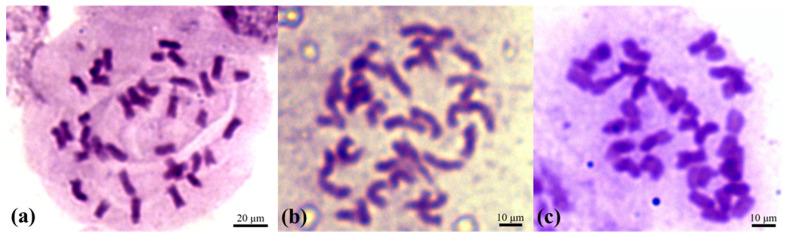
Chromosome observation of CATAS 917 BSs, SSESs, and ABRPs: (**a**) 2n = 36 cells (BSs); (**b**) 2n = 36 cells (SSESs); (**c**) 2n = 36 cells (ABRPs).

After performing triplicate chromosomal analysis by counting the chromosomes in 30 cells, we determined that there was no difference among the BSs, SSESs, and ABRPs. The results confirmed the presence of normal diploid cells with 36 chromosomes (Figure 7) in the tested tissues.

#### 2.4.2. Ploidy Detection of ABRPs

**Figure 8:**
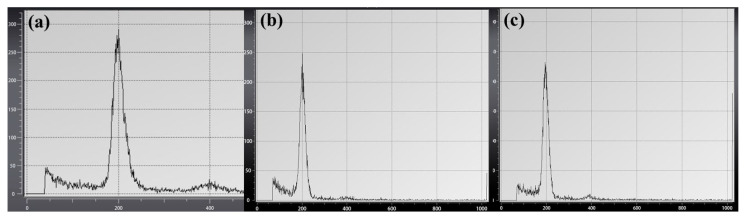
Relative DNA content of three different sources of RT. (**a**) BSs; (**b**) SSESs; (**c**) ABRPs.

Using flow cytometry, the relative DNA content of three plants, each of CATAS 917 BSs, SSESs, and ABRPs, was determined. The results showed that the relative DNA content of the ABRPs was similar to the BSs and SSESs (Figure 8a–c). The histogram revealed the development of identical peak patterns in the three groups at channel 200.

#### 2.4.3. Analysis of ISSR–Polymerase Chain Reaction (PCR) Products from ABRPs

**Figure 9:**
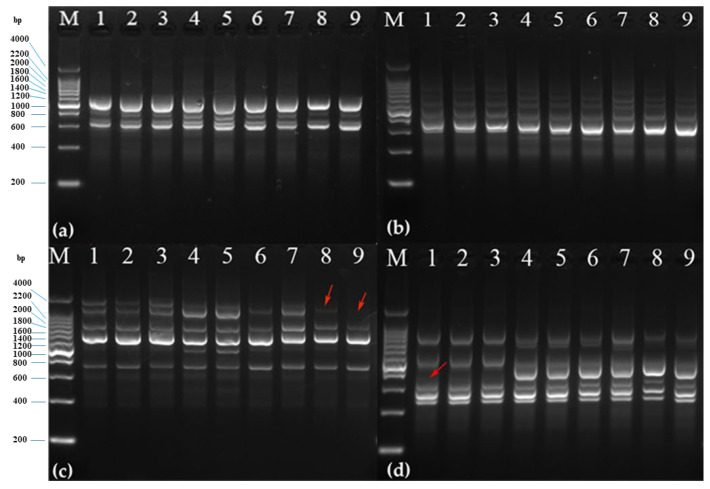
Primers in (**a**–**d**) are the amplified bands of UBC-808, UBC-809, UBC-835 and UBC-840 in BSs, SESSs and ABRPs, respectively. The samples of (**a**–**d**) are: 1~3: SSESs; 4~6: BSs; 7~9: ABRPs; M: DL 200 DNA marker (arrow shows polymorphic bands).

The results showed that there was no variation between BSs and SSESs; however, there was variation among ABRPs (Table 7). In the ABRP genetic diversity assessment, as well as the BSs and SSESs of Hevea using ISSR markers (Figure 9), 488 loci were amplified. Comparing the ABRPs to BSs, only 10 (6.06%) were polymorphic, whereas when comparing ABRPs to SSESs, this number was only 13 (7.74%). The difference in variation can be considered as the least/a poor level of polymorphism.

## 3. Discussion

Due to Hevea’s heterozygous nature and long juvenile period, conventional breeding is highly complicated. The introduction of in vitro breeding techniques became a remedy to overcome these difficulties. However, most of the explants used in Hevea tissue culture techniques are seasonal, creating complexity in the continuation of production [25]. Hence, it became important to establish a nonseasonal explant-based in vitro propagation system with high potential for the seedling regeneration. A successful micropropagation system relies considerably on five factors: explant preparation, the development of aseptic culture, multiplication of propagules, regeneration into a complete plantlet, and acclimatization to greenhouse conditions. Therefore, the most important objective in tissue culturing is to establish an effective protocol including all these stages [26].

In the present study, an axillary-bud-based seedling regeneration system was established for the propagation of in vitro seedlings to overcome complications with seasonal explants. ABs (0~3 d) had a short swelling time from the stem segment of an in vitro seedling, which was inoculated for the AB swelling in the MS media, and this swift AB response exhibited vigorous meristematic ability for further development [27]. It has been reported that, with this potential, ABs can develop into shoots or leaves, and the level of mitogens in endogenous cells will be increased during the development of ABs [28]. Alterations in mitogens, such as cytokinin content, regulate the differentiation and overgrowth of ABs [29,30]. This mechanism is crucial in axillary-bud-based tissue culture techniques.

Usually, AB tissue culture is a two-step process: shoot proliferation and rooting, then obtaining a complete plant [31]. Nevertheless, the current study has been targeted at primary somatic embryogenesis and whole-plant regeneration through callus proliferation from in vitro-induced ABs. To find the best donor plant for AB induction, phenologically different in vitro seedlings were selected. From the results obtained, we confirmed that the phenological impacts were very high on the induction of ABs. Explant age is a crucial factor in the success of plant micropropagation [32,33]. The most suitable phenological stage was the mature period (70.83 ± 7.22), which exhibited the best AB swelling rates compared with the bronze period, color-change period and pale-green period. The use of test tube seedling–stem segments resulted in contamination-free cultures during AB swelling. In vitro culture contamination is considered the major stumbling block in tissue culture experiments.

6-benzyladine is a popular and highly efficient synthetic cytokinin mainly used for crop production. Based on many previous reports, it has the potential to enhance non-meristem differentiation and lateral bud outgrowth [34,35]. Some researchers found that *Acacia auriculiformis* bud growth was greatly influenced by 6-BA (1.5 mg/L) [36]. In this study, the swelling rate of rubber tree ABs could be significantly increased by increasing the 6-BA concentration (up to 85.83% at 2 mg/L), which is consistent with the research findings of [37]. The anatomical results of the ABs showed that the 3 d ABs had meristems with dense cytoplasm, and the callus occurred at the central growth point with meristems. The 10 d ABs had young leaves extending outward. The callus incidence rate of the 3 d ABs (48.72%) was higher than the incidence of the AB callus at 10 d (12.22%). The histological section showed that the swelling times with high callus incidence corresponded to the induction days with vigorous meristems, indicating that the callus incidence was related to the swelling times of ABs.

Callus induction is the major step in the process of culturing AB tissue, and the use of an appropriate medium will be helpful for successful callus formation. Among the nine different basic media checked in the current study, ABs showed remarkable results in obtaining a faster response rate, the highest survival rate, and almost no browning in the basic MH medium. Based on these data, basic MH medium was selected for callus induction, and different combinations and concentrations of 2,4-D, NAA, KT, and sucrose were checked for the optimum combination of callus induction. For callus induction, 2,4-D and NAA are required, and kinetin stimulates the callus proliferation [38,39]. The present study indicated that MH medium supplemented with KT (1.5 mg/L), 2,4-D (1.5 mg/L), NAA (1.5 mg/L), and sucrose 70 g/L was the most suitable combination for inducing a callus from AB swelling. One of the major requirements for plant growth development is a carbon source. The presence of sucrose in the culture medium is the main energy source for the plant cells. The concentration of sucrose in the culture medium may vary in different plant species [40,41]. Recent studies have reported that plants growing in media containing high concentrations of sucrose display higher gains in dry weight and leaf development [42]. Various studies have supported the beneficial effects of these hormones in inducing a callus in Hevea and other plant species [43,44,45,46]. The optimal regeneration potential was obtained in MH media supplemented with 1 mg/L 6-BA, 3 mg/L KT, 0.06 mg/L 2, 4-D, and 0.5 mg/L GA3. A complete plantlet was obtained within 175 d. Shoot elongation and rooting occurred in MH, which contained 0.5 mg/L KT, 3 mg/L GA_3_, and 0.2 mg/L IAA. The development rate was equal with the BSs and SSESs.

The occurrence of genetic and epigenetic changes is the main limitation in the micropropagation of Hevea clones [47]. Thus, it is important to confirm the genetic stability among the plants obtained through somatic embryogenesis [48]. One of the most effective cytogenetic methods for the estimation of genome size and ploidy in plants is flow cytometry. This technique has been used to assess DNA content and ploidy variation among different species, including *Leucocoryneae* [49]. The technique was also used to analyze the ploidy levels of nine Chinese native species of Rosaceae (*Rosa* sp.) [50]. Moreover, flow cytometry is widely used to compare the relationships and origins of different materials within the same genus. In a previous study on wheat, the presence of an extra chromosome was identified based on karyotype analysis [51]. In the current study, chromosome counting and assessment confirmed that ABRPs were diploid, similar to BSs and SSESs. Additionally, the relative genome sizes of ABRPs, BSs, and SSESs were similar, indicating the absence of any abnormality at the genome level for ABRPs.

Molecular tools and their applications are widely implemented on tissue culture seedlings, and their beneficial outputs have been well documented by various researchers worldwide [52,53]. According to [54], it is necessary to estimate the genetic variations caused by the in vitro tissue culture techniques because there is a high possibility for soma clonal variation. In this study, analysis using ISSR markers revealed a low level of polymorphism between the test samples. Similar results were observed in many previous reports [55,56,57]. In the genetic diversity assessments of Hevea ABRPs, BSs and SSESs using ISSR markers, 488 loci were amplified. When comparing ABRPs with BSs, only 10 (6.06%) were polymorphic, while with SSESs, the number rose to 13 (7.74%), indicating a low genetic diversity. Thus, a lower level of difference was observed among ABRPs, BSs, and SSESs. Therefore, we confirmed that AB swelling can be used for the mass regeneration of plantlets through the induction of the callus and somatic embryogenesis pathways without any seasonal barrier.

## 4. Materials and Methods

### 4.1. Experimental Materials

The original material used in the experiment was the SSESs of CATAS 917 of *Hevea brasiliensis*. The primary embryos were obtained from the anthers of *Hevea brasiliensis* and then induced to form secondary embryos. The secondary somatic embryos were proliferated to obtain regenerated plants. The specific method can be found in [6]. The SSESs were cut into small stem segments, and the swelling ABs were stimulated using a medium culture as the explants for the experiment. Therefore, the control seedlings used for genetic stability analysis were SSESs of the same variety and BSs. SSESs were taken from the natural rubber new planting material innovation base of the Rubber Research Institute, Chinese Academy of Tropical Agricultural Sciences (Danzhou, Hainan), and BSs were obtained from the seedling breeding base of the Rubber Research Institute, Chinese Academy of Tropical Agricultural Sciences (Danzhou, Hainan).

### 4.2. Medium and Culture Condition 

The medium used in this experiment is shown in Table 8: 2, 4-D, 3-indoleacetic acid (IAA), NAA, 6-BA, gibberellin A3 (GA_3_), KT, and abscisic acid (ABA), and phytagel, all purchased from Sigma, USA. The Hevea ABs, AB callus, embryonic callus, and SE in M1, M2, M3, and M4 were incubated in the dark at 25 ± 1 °C. ABRPs in M5 were cultured under light at 25 ± 1 °C, a photoperiod of 12 h/12 h (light/dark), and an illumination of 3500–3800 lux.

### 4.3. Experimental Method

#### 4.3.1. Stimulation of AB Swelling

Phenologically different in vitro plantlets (bronze period, color-change period, pale-green period, and mature period) were selected; then, 1 cm-long stem segments were dissected with one bud point. The bud point was inoculated horizontally upward in M1 to study the effect of phenology on AB swelling. Later, to understand the potential of 6-BA on AB swelling, the M1 medium was supplemented with 0, 0.4, 0.8, 1.2, 1.6, and 2.0 mg/L. The culture was maintained at 25 ± 1 °C in the dark, and the swelling rate of the explant was calculated after 3~4 d. The rate of AB swelling (%) was calculated according to the number of swelling ABs/total number of cultured ABs × 100.

#### 4.3.2. Histological Analysis

**Figure 10:**
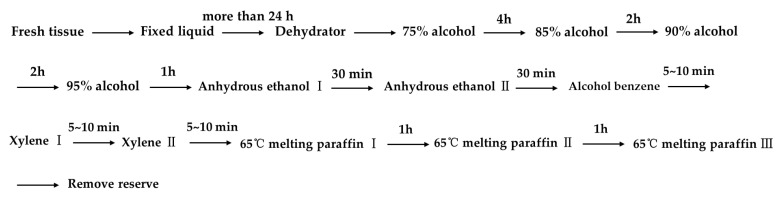
Dehydration waxing process.

ABs, distinct young leaves with mature phenophases and swelling times of 3 d, 6 d, and 10 d were all observed morphologically using a German Leica M165FC fluorescence microscope. Then, the fresh tissue was dewatered and waxed according to the steps in Figure 10.

The melted wax block was embedded in an embedding machine; after treatment, the samples were frozen at −20 °C on a frozen platform; after solidification, the wax block was trimmed and cooled, and then put into the pathology slicer for slicing (4 μm-thick); in the tissue spreader, the slices were unfolded with 40 °C warm water, placed on the slide, dried in an oven at 60 °C, and placed at room temperature for later use.

**Figure 11:**
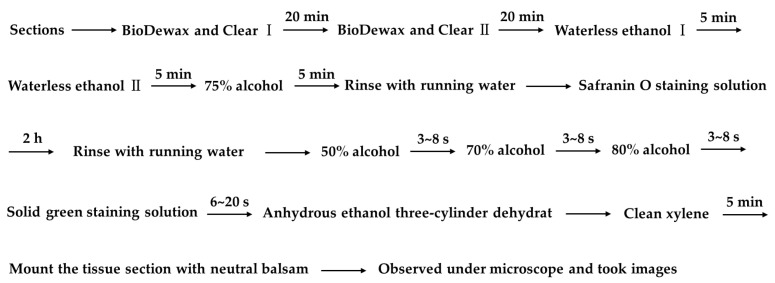
Safranin O-Fast Green staining process.

The prepared slices were stained with Safranin O-Fast Green staining according to the steps in Figure 11.

The production of paraffin sections was completed by Wuhan service biotechnology Co., Ltd. (Servicebio, Wuhan, China).

#### 4.3.3. Callus Induction

After the AB induction during different time periods in M1, the swollen ABs from the 3–4 d groups were selected and transferred into 9 different basic media, such as MH, MS, NN, NLN, N6, B5, GD, SH, and WPM. The effects of basic media on developmental changes were compared. After confirming the effects, the best basal media was chosen for callus induction. To obtain the optimum callus induction level, different concentrations and combinations of 2,4-D, NAA, KT, and sucrose were supplemented with the callus induction medium. The experiment was analyzed based on the L_9_ (3^4^) orthogonal test design of 4 factors and 3 levels. Additionally, the best was selected and termed as M2. Based on the observation of the 25~30 d incubation of AB swellings in M2, the callus induction rate was calculated (callus induction rate (%) = (number of explants inducing callus/number of explants inoculated) × 100).

#### 4.3.4. Induction of Embryogenic Callus

The well-grown yellow granular callus was separated from the browned, hard, and dead callus after 30 d of incubation in M2, and then subcultured in M3 medium for 25~30 d in dark conditions at 25 ± 1 °C.

#### 4.3.5. Induction of Somatic Embryos

The light-yellow embryogenic callus with dense structure induced in M3 was inoculated in M4 medium and cultured in the dark at 25 ± 1 °C for 60~70 d. Subculturing was performed in M4 medium every 25~30 d and photographed every 10 d until the cotyledon was expanded.

#### 4.3.6. Regeneration of Seedling

Once the cotyledons developed and matured in M4 medium, the dicotyledonous embryos were inoculated in M5 at room temperature 25 ± 1 °C and maintained under light conditions of 3500~3800 lux light intensity for a 12/12 h (light/dark) for 40–45 d to obtain the complete seedlings.

### 4.4. Analysis of Genetic Stability

#### 4.4.1. Chromosome Preparation of Regenerated Plant and Mother Plant

The fresh leaves were collected from the bronze-period ABRP, SSES, and BS seedlings of the same cultivar. The chromosome preparation was performed according to [59], with minor modifications. The samples were pretreated in the para-dichlorobenzene-saturated solution (Macklin, Shanghai, China) for 2~2.5 h. Then, we discarded the pretreatment solution and washed using sterile double-distilled water (ddH_2_O) to avoid odor. Then, treatment was performed with sterile ddH_2_O hypotonic solution (1 h) and an absolute ethanol:acetic acid (3:1) solution for 12~20 h. Later, dehydration using 70%, 90%, and 100% ethanol was performed for 5 min at each time and stored in 70% ethanol at −20 °C. The treated plant tissues were washed with sterile ddH_2_O 2~3 times. Prehypotonic treatments were performed with sterile ddH_2_O for 50 min and blot-dried. The plant materials were placed in a centrifuge tube containing enzymatic hydrolysate and hydrolyzed in a water bath at 37 °C for 2~2.5 h. The enzymatic hydrolysate contained 10.5% cellulase + 9% pectinase (which was dissolved in 10 mL phosphate buffer composed of 29.18 g/L NaH_2_PO_4_·2H_2_O, 4.66 g/L Na_2_HPO_4_ · 12H_2_O, and 8 g/L NaCl). We performed the enzymatic hydrolysis process, shaking the centrifuge tube 3~5 times and washing the leaves 1–2 times after completion. Subsequently, ddH_2_O was added, and it remained hypotonic for 40 min. Then, we treated it with anhydrous ethanol and acetic acid (3:1) for 15 min, peeled and crushed the edges of the treated leaves with an anatomical needle, and placed them on a dry and clear slide fixed with an alcohol lamp flame, and then air-dried them. The fixed tissues were stained (modified carbol-fuchsin solution, Solarbio, Beijing, China), then we flushed away the excess dye with a fine flow of water and blow-dried. The number of cells with scattered chromosomes in the regenerated ABs and control plants was counted. We observed 30 of each cell.

#### 4.4.2. Ploidy Detection of ABRPs

The ploidy of the ABRPs was determined by Sysmex Cy-Flow^®^Cube8 flow cytometry. We took 300 mg of fresh leaf from CATAS 917 BSs, using SSESs as a reference, and placed them in a plastic Petri dish. We added 0.5 mL of extraction buffer and chopped them quickly with a sharp blade. We took 30 μm of aperture filter screen, mixed with 1.6 mL (4′,6-diamidino-2-phenylindole) DAPI staining buffer, shook and mixed, and later set it at room temperature for 40 s. Then, we tested it on the machine. Extraction buffer and staining buffer were obtained from Sysmex Corporation (Kobe, Hyogo, Japan). We used flow cytometry connected to computer software (Cubeoffline) to perform the mapping.

#### 4.4.3. DNA Extraction

We took the fresh leaves of CATAS 917 BSs, SSESs, and ABRPs. The DNA extraction was performed according to the commercial kit method (TIANGEN, Shanghai, China). Using an Ultramicro spectrophotometer, we detected the DNA purity and concentration, and we created a 2.0% agarose gel assay; then, DNA at concentrations of 100~300 ng/μL was diluted with ddH_2_O to 30 ng/μL and stored at −20 °C for backup.

#### 4.4.4. ISSR Assay

The eight primers used in the experiment were synthesized by Sangon Biotech, Shanghai, China. The volume of the ISSR amplification reaction system was 20 μL, with 1.5 μL of DNA template (50 ng/μL), primer 0.8 μL, ddH_2_O 7.7 μL, and 2 × mixDNA polymerase 10 μL. The ISSR–PCR reaction procedure was pre-denaturation at 94 °C for 5 min, denaturing at 94 °C for 30 s, anneal at 50 °C for 30 s, extension at 72 °C for 90 s, 45 cycles, 72 °C extension for 7 min, and storage at 4 °C. We obtained 6 μL of amplification product in 2% agarose gel (containing 6 μL of nucleic acid dye), then performed electrophoretic separation. The electrophoresis buffer was 1 × TAE, which we photographed with the Tanon gel analysis system. Image Lab software was used to analyze results.

#### 4.4.5. Data Processing and Analysis

The experiment was repeated three times, the results were expressed as average ± standard deviation of three replicates and means represented by different letters in each column show significant difference at 0.05 level according to Duncan’s t test. Excel 2019 and SAS 8.01 were used to sort out and analyze the data obtained in the experiment. We performed a visual analysis of the data using orthogonal design assistant II (v 3.1) software.

## 5. Conclusions

In conclusion, the focus of this study was to solve the problem of the seasonal restriction of plant sources in *Hevea brasiliensis*, demonstrating the ability of ABs for somatic embryogenesis. Additionally, it provided a valuable reference for establishing an efficient route for the seedling regeneration system through callogenesis and somatic embryogenesis from AB swellings. This method will accelerate the large-scale production of somatic embryo seedlings and transgenic research of RT. In this study, the whole process of plant regeneration was observed and recorded, and the genetic stability of regenerated plants was analyzed. In the future, various studies will be performed to optimize the proliferation efficiency of AB calluses, improve the induction rate of embryogenic calluses, explore the relationship between embryogenic calluses and somatic embryogenesis, and increase the incidence of somatic embryogenesis. Genetic transformation will also be carried out using Agrobacterium tumefaciens to explore the possibility of creating transgenic Hevea using the currently described method. 

## Figures and Tables

**Table 1:** Effect of phenology on AB swelling.

Different Phenological Periods	Average Swelling Rate of ABs (%)
Bronze period	59.72 ± 8.67 ^ab^
Color-change period	35.65 ± 9.85 ^c^
Pale-green period	47.20 ± 8.90 ^bc^
Mature period	70.83 ± 7.22 ^a^

Note: Basic medium Murashige and Skoog (MS) [17]; Average swelling rate of ABs with different letters indicate significant differences (*p* < 0.05).

**Table 2:** Effect of 6-BA concentration on AB swelling.

6-BA Concentration (mg/L)	Average Swelling Rate of ABs (%)
0.0	52.38 ± 4.12 ^cd^
0.4	59.88 ± 2.68 ^bc^
0.8	46.67 ± 5.77 ^d^
1.2	80.24 ± 5.36 ^a^
1.6	65.00 ± 4.33 ^b^
2.0	85.83 ± 5.20 ^a^

Note: Basic medium (MS); Average swelling rate of ABs with different letters indicate significant differences (*p* < 0.05).

**Table 3:** Effect of the time of AB swelling on callus formation.

AB Swelling Time	Average Incidence of Callus (%)	Explant Swelling Status
3 d	48.72 ± 4.44 ^a^	High callus growth with a granular ball shape
6 d	54.29 ± 9.90 ^a^	Irregular callus morphology
10 d	12.22 ± 10.72 ^b^	There was almost no callus differentiation—manifested as bud elongation
Differentiation young leaves	21.75 ± 6.14 ^b^	Callus growth was small and irregular

Note: Basic medium (MH); Average incidence of callus with different letters indicate significant differences (*p* < 0.05).

**Table 4:** Effect of basic medium on AB callus induction.

Basic Medium	Average Survival Rate (%)	AB Growth Status
MH	91.90 ± 7.33 ^a^	Fast developmental response (DS), almost no browning, surface protrusion, and tendency of differentiation
MS	87.74 ± 2.15 ^a^	Moderate DS, almost no browning, overall expansion without elongation
NN	31.19 ± 7.84 ^d^	No evident DS and heavy browning
NLN	67.98 ± 4.80 ^b^	Slow DS, considerable browning, slight swelling, elongation, drying, and hardening
N6	66.39 ± 3.76 ^b^	Slow DS, considerable browning, expansion, drying, and hardening
B5	61.11 ± 9.62 ^b^	Slow DS, more severe browning, puffing, and drying hard
GD	67.59 ± 8.82 ^b^	Slow DS, considerable browning, expansion and elongation, drying and hardening
SH	44.81 ± 5.01 ^c^	Slow DS, severe browning, expansion and elongation, drying and hardening
WPM	87.37 ± 1.59 ^a^	Fast DS, partial elongation, drying and hardening

Note: Average survival rate with different letters indicate significant differences (*p* < 0.05).

**Table 5:** Effect of different hormone concentration combinations on AB callus formation.

Test Number	2, 4-D (mg/L)	NAA (mg/L)	KT (mg/L)	Sucrose (g)	Average Incidence of Callus (%)	Callus Swelling Status
1	1.5	1.5	1.5	70	56.55 ± 6.27 ^a^	Slight browning with a pronounced reaction and a high amount of callus growth
2	1.5	0.75	0.75	46	22.41 ± 2.51 ^c^	The browning was considerable, with a small amount of callus
3	1.5	3	3	105	0.00 ± 0.00 ^e^	Heavy browning with little or no callus occurring
4	0.75	1.5	0.75	105	0.00 ± 0.00 ^e^	Slight browning with little or no callus occurs
5	0.75	0.75	3	70	23.15 ± 1.60 ^c^	Slightly browning, with large callus swelling
6	0.75	13	1.5	46	33.81 ± 7.87 ^b^	Heavy browning, with few calluses occurring
7	3	1.5	3	46	11.20 ± 1.25 ^d^	Slightly browning, with large callus swelling
8	3	0.75	1.5	105	33.13 ± 4.47 ^b^	More severe browning and low callus swelling
9	3	3	0.75	70	22.41 ± 2.51 ^c^	Slight browning, with less callus swelling

Note. Basic medium (MH); Average incidence of callus with different letters indicate significant differences (*p* < 0.05).

**Table 6:** Intuitive analysis of callus induction rate in orthogonal experimental design.

Analytical Indicators	K Value	2,4-D (mg/L)	NAA (mg/L)	KT (mg/L)	Sucrose (g/L)
Average callus induction rate	K1	25.927	22.223	40.740	33.333
	K2	18.517	25.923	14.813	22.220
	K3	22.220	18.517	11.110	11.110
	R	7.410	7.406	29.630	22.223

Note: K1, K2 and K3 are the average values of each treatment under the same factor; R is the range of each level of data under the same processing conditions.

**Table 7:** Base sequence amplification of the eight ISSR primers.

Primer Name	Primer Sequence	Number of Amplified Bands								
		BSs 1	BSs 2	BSs 3	SSESs 1	SSESs 2	SSESs 3	ABRPs 1	ABRPs 2	ABRPs 3
UBC-807	(AG) 8T	8	8	8	8	8	8	8	6	6
UBC-808	(AG) 8C	4	4	4	4	4	4	4	4	4
UBC-809	(AG) 8G	7	7	7	6	6	6	6	6	6
UBC-834	(AG) 8YT	7	7	7	7	7	7	7	7	7
UBC-835	(AG) 8YC	7	7	7	7	7	7	7	6	5
UBC-836	(AG) 8YA	8	8	8	8	8	8	8	7	7
UBC-840	(GA) 8YT	7	7	7	8	8	8	8	6	6
UBC-841	(GA) 8YC	7	7	7	8	8	8	8	8	8
Total	488	55	55	55 (165)	56	56	56 (168)	56	50	49 (155)

**Table 8:** The medium used in the regeneration of rubber tree CATAS 917 ABs.

Medium	Composition
The culture medium for AB swelling (M1)	MS + 2 mg/L 6-BA + 30 g/L sucrose + 2.2 g/L phytagel, Ph 5.8 (the same below)
Callus induction medium (M2)	MH + 1.5 mg/L 2,4-D + 1.5 mg/L NAA + 1.5 mg/L KT + 70 g/L sucrose + 2.2 g/L phytagel
Embryogenic callus induction medium (M3)	MH + 20 g/L sucrose + 2 g/L activated carbon + 2.2 g/L phytagel
Somatic embryo differentiation and maturation medium (M4)	MH + 0.5 mg/L 6-BA + 3 mg/L KT + 0.02 mg/L NAA + 0.5 mg/L GA_3_ + 1 mg/L ABA + 70 g/L sucrose + 2.2 g/L phytagel
Plant regeneration medium (M5)	MS + 0.05 mg/L KT + 3 mg/L GA_3_ + 0.02 mg/L IAA + 2.2 g/L phytagel

Note: MH was based on MS medium, containing a large number of elements of 4/5, and the rest of the components were the same. The M3 medium was modified using [58]. M2, M4 and M5 media were modified using [6].

## Data Availability

All available data are contained within the article.
